# Monthly Summary

**Published:** 1884-09

**Authors:** 


### MONTHLY SUMMARY.

---

HOW TO MAKE A PERFECT FITTING PLATE.—In the course of our professional life it is a good plan to pause now and then to re-examine our accepted methods and see if perchance they may be improved. Mankind are very prone to accept that which is, as being all that is needed. All this looking ahead to discover new fields, new ideas, and new methods is well enough, and leads to progress. For the present we will leave that to others and spend a few moments in examining present methods, as some times real progress is not made because of imperfections in present methods, which must be the foundation upon which future progress will have to be based.

It has been, and is now, the custom to make air chambers in upper plates; the object of which is the better retention of said plates in the mouth. It is evident that the plate is held in position by the pressure of the atmosphere upon one side of it, the air being pressed out from the other side by the exact coaptation of the surface of the plate to the surface of the mouth, the pressure being just in proportion to the surperfices of the plate. Now I am utterly at a loss, when I seek for a law of natural philosophy that causes a plate to adhere with greater firmness, when it has an air chamber under it, of even one tenth its superficial area. The natural tendency of such an air chamber must be to reduce the pressure. If the pressure is too great, then an air chamber may be of benefit in reducing the pressure. I have, however, never heard any one claim that the pressure would be too great. My observation has been that in soft mouths the cauity becomes entirely filled by the soft tissue—this is caused by the greater pressure upon the surrounding tissues—thus forcing them into the cavity, and producing congestion and inflammation, and thereby becoming an instrument of torture, rather than an aid to the better retention of the plate. Believing them to be of no advantage whatever, I have discarded air chambers entirely, both in hard and soft mouths, and have found my work gives better satisfaction than when I followed the beaten path. I never yet have found a brother practitioner who could give a better reason for their use than that the authorities all recommended them. Try the following experiments:

Take two pieces of smooth glass of eqeal size, moisture them and press them together, and notice the tenacity with which they adhere to each other. Then grind a cavity in one of them, proportioned to the size of the glass, as the air chamber is to the plate; press them together in the same manner, and see if they adhere with greater tenacity then before. If they do not, then the reason usually given for the use of air chambers be relegated to the museum of things of the past which mark as stepping stones, our progress. During the past seven years I have tried all the various methods of fitting plates that have come to my notice, and as a result of such experimentation have adopted the following method, which at least has given good results in all cases where the patient had teeth articulated over the whole grinding surface of the molars. If my method affords the same aid to others that it has to me, I shall feel amply repaid for represent it to you to-day.

We all know that plaster, if used with water alone, will warp more or less, and a misfit of greater or less degree is the result. I have tried various means to avoid warping. I now use one and a half teaspoonsful of potash alum to the pint of water and use the solution in mixing plaster. I use this solution in all my processes with plaster and find it answers a good purpose. In taking impressions I use plaster as thick as I can work it, and in taking the second impression for the model, I mix it just thin enough to run into and fill perfectly the impressions made in the first cast. I believe the secret of working plaster so as to avoid warping lies in stirring the mixture as little as possible, and using it as stiff as will allow of a clear and perfect impression. After taking the first impression, take a wax-knife or large spoon excavator, and scrape the impression, from the maxillary tuberiosity on the left, around the alveolar process to the maxillary tuberosity on theright, about the thickness of heavy writing paper. Then scrape over the incisive foramen, along the palate process of superior maxilla and of the palate, to the posterior nasal spine. By feeling in the mouth with the fingers you will ascertain the distance to the hard surfaces connected with these parts. All the hard surface must be scraped. The amount of scraping to be done will be determined by the character of soft tissue. If very soft, scrape the thickness of blotting paper; if hard, the thickness of thin writing paper will be sufficient. Now, place the impression in water, and saturate it, using neither varnish nor oil. Mix a little Spanish brown with your plaster, to change the color, so that you can tell when you cut down to the model. By the difference in color you will find the separate as good as when varnish and oil have have been used. As soon as you have your model ready, turn it over and scrape it on both sides of the palate process over the posterior palatine foramen and groove anteriorly about one-half inch and to the edge of the alveolar process. It needs to be scraped so that it will bear hard upon the soft tissues. Some mouths require as much as one tenth of an inch. By means of this scraping your plate will rest hard upon soft tissues and after a few days wearing will bear upon the hard tissues equally, thus preventing the rocking and falling down of the plate. I sometimes scrape the model under the lips and next to the cheeks so that it may press firmly at these points.

In the lower plate, after the impression, I scrape the bottom of the impression, when the teeth have been long extracted, about the thickness of No, 60 tin foil, and after I get my model I scrape the labial, buccal and lingual surfaces about the thickness of thin writing paper. When about to take the bite, I say nothing to the patient of my intentions, but place my wax plates in the mouth and order the mouth closed, and at the same direct them to perform the act of deglutition; at that instant I press the wax together. After the bite is made and wax removed from the mouth, I have the patient push the chin forward and bite, then draw the chin back and bite again; by doing this, I am enabled to tell, when I get the model in the articulator, whether the bite is correct or not.

By following this plan, I have only had two wrong bites in the past five years. In articulating the teeth, I set them as far under the ridge as possible. If gum teeth will not go under, I substitute plain teeth, and will rather sacrifice the beauty of a joint at the bicuspids and molars, than not have them go under the ridge. The leverage must be overcome, and I know of no better way than to set the teeth under and as near where the natural teeth were as possible, and I consider it far better to use plain teeth as bicuspids and molars than to let the teeth project from the ridge. I use plain teeth with pink rubber for gums, when the patient will consent, and thereby make the teeth more serviceable in masticating food. Gum teeth are well enough in their place, but I would dispense with them entirely if permitted. They remind one of soldiers on dress parade, and cause too great a similiarity of expression.

In grinding the teeth on the model, for old people, I grind off the cusps of the bicuspids and molars slightly, and also square the front teeth to make them look as if worn by age. I make them articulate so that the back teeth will strike first and leave a space the thickness of blotting paper between the front teeth. After they are worn a few days you will find them close together, and the front blocks will not be crowded off the plate. I am confident that if you will try this plan, you will be pleased with the results, and your patients will meet you with a smile instead of that care-worn and discouraged look so often seen.—*Archives of Dentistry.*

---

ANTISEPTIC ABSORBENT SPONGE.—Mr. Sampson Gamgee showed before the Medical Society of London, April 21st, an artificial antiseptic sponge of his invention. A small capsule, containining eucalyptus or other antiseptic, was enclosed in absorbent cotton; outside of this was a layer of cocoanut fibre, and outside of this more absorbent cotton-wool; the whole being enclosed in gauze. When about to be used the capsule could be broken by a blow of the fist, and the absorbent cotton became permeated with the antiseptic. He said these sponges could be made at a very trifling cost, and hoped they would come into use as a cheap substitute for ordinary sponges; they possessed this great advantage, that when required for use they were certain—however long they might have been kept—to be antiseptic: and, being so cheap, they might always be destroyed after being used.—*Canada Medical and Surgical Journal.*

---

TREATMENT OF EPISTAXIS.—An ingenious application of the condom in the treatment of epistaxis was suggested by the late Prof. McDowell. For the purpose of arresting the hemorrhage the condom is tied to the end of a small flexible catheter and a piece of rubber tubing, with a clamp, connected with the catheter. The condom is then lubricated and introduced into the nostril from which the bleeding proceeds, and when in place inflate with air or water as desired.

---

CHLORAL HYDRATE AS A VESICANT.—It is stated that powdered chloral hydrate, sprinkled upon adhesive plaster which is then sufficiently heated to cause it to adhere to the skin, and immediately applied to the surface where a blister is desired, will as effectually accomplish the purpose in ten minutes as a cantharidal plaster will in six hours. The pain produced by it is but slight.

